# Reflective practice and psychotherapy case experience of Specialty Doctors and Associate Specialists (SAS) working in psychiatry: UK-wide survey

**DOI:** 10.1192/bjb.2022.96

**Published:** 2024-02

**Authors:** Alina Vaida, Masud Awal

**Affiliations:** 1 Birmingham and Solihull Mental Health NHS Foundation Trust, Birmingham, UK; 2 Coventry and Warwickshire Partnership NHS Trust, Warwick, UK

**Keywords:** Education and training, SAS psychiatrists, reflective practice, psychotherapy training, Balint groups

## Abstract

**Aims and method:**

To survey nationwide opportunities for Balint-type and reflective support group participation and psychotherapy training among doctors classified as Specialty Doctors and Associate Specialists in psychiatry (‘SAS psychiatrists’) and the professional benefits and barriers to access.

**Results:**

Approximately 9% of SAS psychiatrists responded, from all UK regions. A minority reported participating in a Balint-type group (27.3%) or reflective practice/support group (30.9%), and only 6.5% were not interested in participating. Although 44.8% planned to see a psychotherapy case, most reported barriers, particularly time constraints, job plans and lack of support. The 22.1% who reported already gaining psychotherapy case experience reported many benefits, including becoming a better listener (84.8%), more empathetic (81.2%), enjoying work more (78.8%) and overall becoming a better psychiatrist (90.9%).

**Clinical implications:**

The reported interest in Balint group and psychotherapy training opportunities exceeded existing provision; psychotherapy case experience benefited professional development and self-reported clinical capabilities. Healthcare trusts and boards need to consider more actively supporting SAS psychotherapy training and reflective practice.

Many doctors working within psychiatry are neither consultants nor trainees. They are referred to as Specialty Doctors and Associate Specialists in psychiatry^1^ (‘SAS psychiatrists’) and they form an essential part of the medical workforce.

Specialty doctors are required to engage in continuing professional development activities (CPD). There is no formal requirement for SAS psychiatrists to complete psychotherapy training. However, if they intend to apply for higher training, doctors must satisfy the core psychiatry competencies, including experience in psychotherapy and Balint groups.^2^ To pursue a Certificate of Eligibility for Specialist Registration (CESR) as a means for an SAS psychiatrist to be eligible to apply for substantive consultant jobs, there is a requirement to deliver psychotherapy in two different modalities.^3^ In addition, SAS psychiatrists may pursue psychotherapy interests for their career development and well-being.

Research suggests that seeing psychotherapy cases benefits psychiatric trainees’ professional development and clinical capabilities.^4,5^ However, there is a lack of such evidence for SAS psychiatrists, whether this experience is for CESR applications or professional development. There is also evidence of the benefits for medical students of seeing psychotherapy cases as part of a psychotherapy scheme or being part of Balint groups, as it increases students’ knowledge of the doctor–patient relationship.^6^

One of this paper's authors ran a Balint-type group for SAS psychiatrists working in Birmingham and Solihull Mental Health Foundation Trust (BSMHFT) and was impressed by the doctors’ interest in and commitment to the group and their embracing of psychologically informed practice, and another author had provided frequently requested psychotherapy training support to the trust's CESR training programme, which continues to be in demand. However, we were not aware of literature regarding whether such interest was mirrored nationwide.

A literature search on the topic (on PsycInfo, MEDLINE and Embase) did not return any relevant papers. In a comprehensive paper about psychotherapy training needs for psychiatrists and aspirations for better training, psychiatrists who were not in formal training roles were mentioned as providing clinical cover for psychiatry trainees while the latter met their psychotherapy competencies.^7^

Previous research about Balint-type groups in medical education had outlined several benefits, including improved patient–doctor communication, empathy, reflection and reduced burnout.^4,5^ Usually, Balint-type groups are offered to groups of doctors working in the same specialty and with similar experiences (such as core trainees, general practitioners and medical students). ‘Reflective practice’ is a broader category described in the literature that can include Balint-type groups but also encompasses a wider range of activities (such as team reflection or *ad hoc* reflection coupled with teaching days or supervision groups).

In the light of existing evidence suggesting benefits to trainees, importance to professional development and increasing perceived need for reflective space and well-being support during the post-COVID era, we thought it important to investigate whether such benefits, interests and corresponding availability of Balint-type groups, reflective practice groups and other psychotherapy training opportunities was evident for SAS psychiatrists in the UK, and whether the available opportunities met SAS psychiatrists’ developmental needs. We did this by surveying SAS psychiatrists nationwide, aiming to map out the current provision, opportunities, interests and benefits of such experiences.

## Aim

To investigate SAS psychiatrists’ opportunities for Balint-type groups, reflective support groups, psychotherapy training opportunities and psychotherapy case experience, as well as the professional benefits and barriers to access nationwide, for which there is a lack of existing literature or established framework.

## Method

An online questionnaire was sent to UK-wide SAS psychiatry doctors, with support from the Royal College of Psychiatrists (RCPsych) Specialty Doctors’ and Associate Specialist Committee (SASC) through email and online social media groups and platforms catering to SAS psychiatrists. The survey enquired about their location, work experience, future career plans, availability of Balint-type and reflective practice groups, psychotherapy opportunities and available support.

There was a maximum of 18 questions depending on the responses given; there were questions about participants’ experience in delivering psychotherapy and anticipated difficulties, where relevant. Most questions included free-text comment boxes and an additional comments box at the end of the questionnaire.

The questionnaire was open for responses between 20 February and 20 April 2021. The numerical data were analysed using Microsoft Excel and we also reported on the qualitative data from the comments sections. The data were subsequently analysed using IBM SPSS statistics predictive analytics software (version 22 for Windows), identifying the predictive value of independent variables.

## Results

### Demographic data

One hundred and fifty-four doctors completed the questionnaire from across all UK nations, estimated to constitute approximately 9% of SAS psychiatry posts. (To estimate a response rate we compared the respondent numbers with numbers of SAS posts identified in the 2015 RCPsych's census; however, as large number of SAS posts are currently vacant the true response rate is likely to be higher). Apart from one stem question, the questions did not require a mandatory response to progress. Therefore, some respondents did not respond to all the questions, but the number of non-responses to each question was relatively small. Twenty-three participants (14.9%) had missing data. Overall, 1.8% of all values were missing. The percentage reported below for each question refers to the total number of respondents for that question, and the number of non-responses is also given where applicable.

We obtained responses from all major UK regions. Most responses (*n* = 109; 71.7%) were from England, 26 responses (17.1%) were from Scotland, 9 responses (5.9%) from Wales and 8 responses (5.3%) from Northern Ireland. Two respondents skipped this question.

The time spent in an SAS role varied widely, between 17 months (*n* = 17; 11.0%) to over 20 years (*n* = 8; 5.2%). Most respondents had spent between 8 and 12 years (*n* = 35; 22.7%) in an SAS role, 34 respondents (22.1%) had spent 4–7 years, 31 respondents (20.1%) had spent 1–3 years and 29 (18.8%) had spent 13–19 years. There were no missing data for this question.

Regarding future career plans, most respondents (*n* = 87; 58.8%) had plans to pursue higher training or the CESR pathway (53 CESR, 22 training, 12 considering both), 46 (31.1%) were unsure and 15 (10.1%) had no plans to change their career role. This question had 148 responses out of 154 respondents (96.1%).

### Availability of reflective practice and Balint groups

There were separate questions about participation in and availability of Balint and reflective groups.

Of 143 (92.8%) responses, 39 (27.3%) reported being part of a Balint-type group, whereas 67 (46.9%) said they would be interested in joining one but none were available; 21 (14.7%) reported that a group was available but they did not participate; 2 (1.4%) said that no Balint group was available and they would not be interested in participating; 14 (9.8%) said they ‘didn't know’ if a Balint group was available..

Of 152 (98.7%) respondents, 47 (30.9%) were part of a reflective practice or support group, whereas 72 (47.3%) reported that they were interested in joining but none were available; 8 (5.3%) responded that a group was available but they did not participate; 8 (5.3%) said there was no reflective group available and they would not join if one were available; 17 (11.2%) said that they ‘didn't know’.

Combining the responses to the two questions above, the number of respondents who said they were not participating and not interested in either a Balint group or a reflective group totalled 10 (6.5%).

This meant that there were 1.7 times more respondents interested in joining a Balint group (67) than respondents who were already part of a Balint group (39), and there were 1.5 times more respondents interested in joining a reflective practice group (72) than respondents who were already part of a reflective practice group (47).

### Access to psychotherapy cases and supervision

According to the SAS Charter, local education providers should ensure that doctors have access to an SAS tutor (where available).^8^ There is no specific requirement regarding access to a psychotherapy tutor. In our survey, 110 (71.4%) respondents had access to an SAS tutor and 39 (25.5%) reported having access to support from a psychotherapy tutor. There were no missing data for these two questions.

Around half of respondents indicated they did not have access to the information and guidance they needed regarding psychotherapy opportunities (74 respondents, 48.7%), with only 43 (28.3%) thinking they did; 35 (23.0%) respondents chose a ‘neither agree nor disagree’ answer. This question was answered by 152 respondents.

**Fig. 1:**
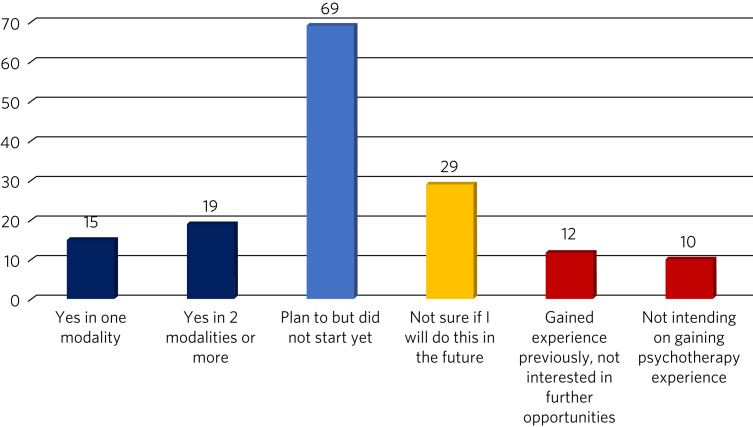
Responses to the survey question ‘In your current role, did you gain experience in delivering psychotherapy?’.

Only 34 respondents (22.1%) reported gaining experience in at least one psychotherapeutic modality; 12 (7.8%) had gained experience previously but were not interested in future opportunities, and 10 (6.5%) said they did not intend to pursue this. The greatest percentage of respondents (*n* = 69; 44.8%) was comprised of doctors who were planning to start; 29 (18.8%) said that they were not sure whether they would do this in the future (Fig. 1).

**Fig. 2:**
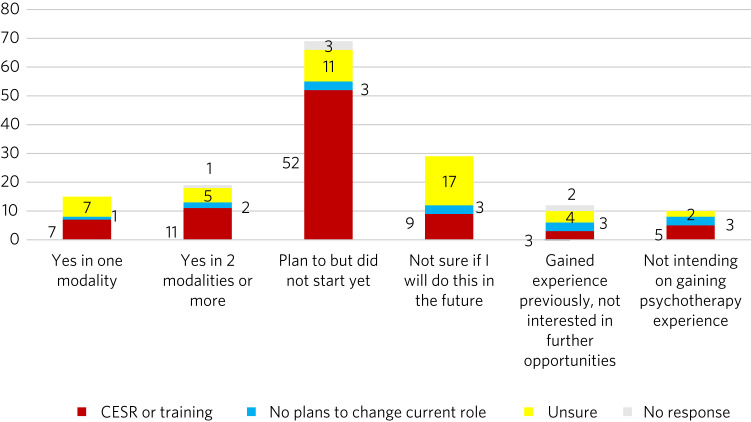
Responses to the survey question regarding experience of delivering psychotherapy, subdivided by future career plans.

We broke data down further by looking at the future career aspirations of the groups above. We found that each subgroup was formed of a mixture of doctors with different career aspirations, and the groups of SAS psychiatrists who managed to deliver psychotherapy or were pursuing this were not only formed of the doctors planning to apply for CESR or higher training (Fig. 2).

The statistical analysis (multinominal logistic regression) revealed that SAS psychiatrists who were intending to apply for the CESR pathway or higher training were significantly more likely to have delivered psychotherapy in two modalities (odds ratio OR = 0.015, meaning 98.5% more likely) and were more likely to plan to start psychotherapy cases (OR = 0.008, meaning 99.2% more likely) compared with those with no such plans to change their current roles.

There was no statistical difference between doctors with or without access to a psychotherapy tutor or an SAS tutor in terms of their psychotherapy experience. There was also no statistical difference between doctors with more years of experience in the role and their psychotherapy experience.

The respondents’ answers to this stem question about their psychotherapy case experience led to different branches of questions for respondents depending on their answers, as below.

### The experience of SAS psychiatrists who managed to deliver psychotherapy under supervision (*n* = 34; 22.1%)

**Fig. 3:**
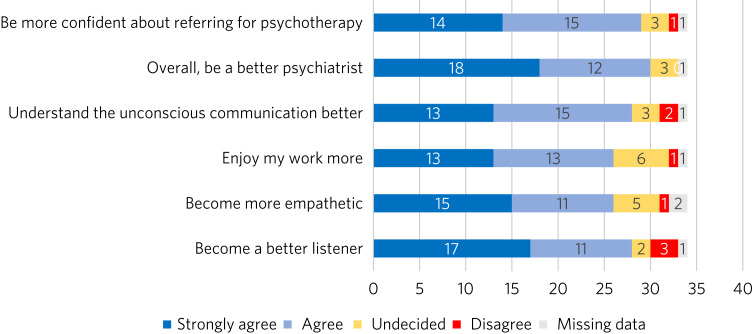
Responses to the survey statement ‘Experience of delivering psychotherapy helped [me] to:’.

Most of the respondents who had delivered psychotherapy agreed or strongly agreed that it had helped them become a better listener (84.8%, 28 out of 33 respondents), become more empathetic (81.2%, 26 out of 32 respondents), enjoy work more (78.8%, 26 out of 33 respondents), understand unconscious communication better (84.8%, 28 out of 33 respondents), overall be a better psychiatrist (90.9%, 30 out of 33 respondents) and be more confident about referring for psychotherapy (87.8%, 29 out of 33 respondents). A very small number of doctors disagreed with any of the statements, and no one selected ‘strongly disagree’ for any of the questions (Fig. 3).

The majority of the respondents who had delivered psychotherapy reported good/excellent experiences regarding the quality of supervision (21 out of 32 respondents, 65.6%), had support in identifying suitable cases (18 out of 32 respondents, 56.3%) and felt they had received the required quality of teaching or training in psychotherapy before starting (16 out of 31 respondents, 51.6%). We also asked about receiving support for completing *non*-psychotherapy requirements for CESR. We noted that this question did not apply to 37.5% of respondents (12 out of 32 respondents); however, only 8 (25%) respondents rated the support as good and none as excellent. These questions had between one and three missing answers out of the total of 34.

Looking at the future career plans of this subgroup, 18 (54.5%) said that they were planning to apply for CESR or higher training, 12 (36.4%) said that they were unsure and 3 (9.1%) had no plans to change their career path (one did not answer).

Participants were asked about barriers in accessing psychotherapy experience; out of 34 respondents, the majority (*n* = 27; 79.4%) listed at least one barrier, with some listing more barriers (up to 6).

The most frequently reported barriers were time constraints, reported by 18 doctors (52.9%), followed by difficulty accessing supervision, reported by 14 (41.2%). Thirty-one responses were received regarding supervision: most stated that the participant was supervised by a medical psychotherapist (*n* = 13; 41.9%), followed by a psychotherapist (*n* = 9; 29%) and then a clinical psychologist (*n* = 8; 25.8%).

The most frequently delivered form of psychotherapy was CBT, reported by 14 out of 34 respondents (41.2%), followed by psychodynamic (*n* = 12; 35.3%) and interpersonal psychotherapy (*n* = 5; 14.7%).

Looking at this subgroup's participation in a Balint or reflective practice group, we noted that 15 (44.1%) respondents were part of either a Balint or reflective group, 15 (44.1%) would be interested if one were available, 2 (5.9%) said that they were not interested in joining and 2 (5.9%) ‘didn't know’ if such a group was available.

The free-text comments suggested that psychotherapy experience might be more accessible if it were part of the job plan and if they had access to a supervisor. One participant suggested having a dedicated psychotherapy supervisor for CESR candidates. In total, 23 of the 34 (67.6%) responded to questions about psychotherapy courses; 7 had attended a long psychotherapy course of up to a year; 8 had a psychotherapy qualification, such as an MSc or diploma, and 8 had attended short courses (between 2–5 days); 7 out of these 23 respondents said that they had attended more than one psychotherapy course.

### The experience of SAS psychiatrists who were planning to start psychotherapy but had not started yet (*n* = 69; 44.8%)

In this subgroup, out of the 66 respondents to the question about future career plans (3 left this question blank), 52 (78.8%) said that they planned to apply either to CESR or higher training, 11 (16.7%) were unsure and 3 (4.5%) were remaining SAS by choice. Out of 68 respondents (one did not answer), 19 (27.9%) said that they did not have previous experience of psychotherapy; 13 (19.1%) had had such experience more than 10 years ago, with the remaining doctors reporting such experience between less than 1 year and up to 10 years ago.

Regarding difficulties that got in the way, 2 (2.9%) out of 68 respondents (one response left blank) said there were ‘none they can think of’, and the majority selected at least one difficulty, with a mean of 2.8 difficulties. The difficulty chosen most frequently was ‘not part of job plan’, reported by 48 (70.6%) out of 68 respondents, followed by ‘time constraints’ and ‘difficulties in accessing supervision’, with 40 (58.8%) responses for each. Of the 69 respondents, 18 (26.1%) managed to identify a supervisor, whereas the majority did not (*n* = 45; 65.2%) or were not sure (*n* = 6; 8.7%). In total, 29 (42%) said that they had completed a psychotherapy course, mostly of short duration (up to 5 days), with 3 (4.3%) completing a longer course of up to a year or a psychotherapy qualification.

From the free-text comments at the end of this section of the questionnaire, the most common theme was about psychotherapy opportunities and reflective groups not being available for SAS doctors or about them only being available for trainees; one doctor wrote that they had to change their job to access psychotherapy supervision in another trust for their CESR application. Pressure on services and staff was also highlighted as a barrier for SAS doctors, with one respondent commenting ‘Currently no one has the time or energy for the luxury of learning and developing interests, though recognise their importance to keep our motivation and spirits alive [ … ] We all know these things would be helpful but taking time to participate will further increase the fear of serious adverse incidents because services are stretched’.

## Discussion

### Summary

- The number of respondents was relatively low, at about 9% of the approximated SAS psychiatrist numbers, but the responses were spread across the four nations of the UK, suggesting representation from all major regions.
- More than half of respondents (58.8%) said that they had plans to pursue higher training or the CESR pathway, a higher percentage than reported in a General Medical Council (GMC) survey^9^ for SAS doctors of all specialties, where a quarter reported such plans; the GMC survey did not enquire about plans to pursue higher training, so comparing like for like, 42.2% from our cohort said that they considered either CESR or further formal training (65 out of 154). A possible explanation is that the SAS psychiatrists who have plans to pursue CESR or training were more likely to respond to our survey, as they must meet the psychotherapy requirements to pursue their future career plans.
- Most respondents were part of or interested in being part of a reflective group; only 6.5% said they were not participating in or not interested in either a Balint group or a reflective practice group.
- Respondents who delivered psychotherapy reported enjoying many benefits, including becoming a better listener (84.8%), more empathetic (81.2%), enjoying work more (78.8%), understanding the unconscious communication better (84.8%), overall being a better psychiatrist (90.9%) and being more confident about referring for psychotherapy (87.8%). These benefits have a bearing on SAS doctors’ professional development and may lead to more psychologically informed reflective clinical practice. The benefits would likely extend to their patients’ care, including generalisable and frequently relevant consultation skills.
- Many barriers got in the way of delivering psychotherapy. For the doctors who managed to see psychotherapy cases, the most frequently reported was ‘time constraints’; for those who planned to start psychotherapy, the most frequently reported main barrier was that it was ‘not part of job plan’. In contrast, a similar survey aimed at psychiatry trainees had found that the main barriers identified were ‘patient factors (such as ‘drop out’)’ and ‘delay in patient allocations’.^10^
- Respondents intending to apply for the CESR pathway or higher training were more likely to have delivered psychotherapy in two or more modalities and to plan to start psychotherapy cases.
- There was no statistical difference between respondents with or without access to a psychotherapy tutor, in terms of psychotherapy experience. One explanation may be that, even though some SAS psychiatrists have access to a psychotherapy tutor, they may not be able to help with supervision and/or cases if that is not part of the psychotherapy tutor's agreed role and the SAS doctor may still face challenges regarding time constraints and psychotherapy not being part of their job plan.

### Implications

Overall, SAS psychiatrists who delivered psychotherapy reported benefits on many levels, making a strong case that it develops their clinical capabilities, which may facilitate psychologically informed care. The results indicate that both interest in psychotherapy training and opportunities for reflective practice/Balint group participation significantly outstripped available options and support. As these groups are not mandatory requirements for CESR application, the interest expressed (including among those reporting to be remaining SAS by choice) suggests that SAS psychiatrists value these opportunities for their recognised professional developmental and clinical benefits; these include peer support, understanding doctor–patient interactions and having a space to reflect on the emotional impact of clinical work.

Although some barriers mirrored those previously reported for trainees (difficulties accessing supervision and cases), others primarily related to SAS workload (not being part of their job plan, time constraints and service pressures) and lack of support (with trainees prioritised). This may highlight a potential concern, given that the SAS Charter covers CESR-related support and advocates appropriate Supporting Professional Activities (SPA) time. Our results suggest that healthcare trusts and boards need to consider more actively supporting SAS psychotherapy training and reflective practice groups and including them in job planning for those receiving, delivering and supporting these valued experiences.

## Data Availability

Copies of the survey and the data collected can be supplied upon reasonable request to the corresponding author. When sharing responses, location and any identifiable data will be removed as the completion of the survey was done within anonymity limits.

## About the authors

**Alina Vaida** is Consultant Psychiatrist in Medical Psychotherapy working in Liaison Psychiatry and psychotherapy tutor with Coventry and Warwickshire Partnership NHS Trust, UK. When the survey was designed and the data was collected she worked with the Specialist Psychotherapies Service of Birmingham and Solihull Mental Health NHS Foundation Trust, Birmingham, UK. **Masud Awal** is Consultant Psychiatrist in Medical Psychotherapy and psychotherapy tutor with the Specialist Psychotherapies Service of Birmingham and Solihull Mental Health NHS Foundation Trust, Birmingham, UK. Dr Awal is also the regional psychotherapy tutor and medical psychotherapy training programe director for West Midlands, UK.
